# What kind of a problem is loneliness? Representations of connectedness and participation from a study of telepresence technologies in the UK

**DOI:** 10.3389/fdgth.2024.1304085

**Published:** 2024-02-19

**Authors:** Gemma Hughes, Lucy Moore, Megan Hennessy, Tony Sandset, Elian E. Jentoft, Marit Haldar

**Affiliations:** ^1^ School of Business, University of Leicester, Leicester, United Kingdom; ^2^ Nuffield Department of Primary Care Health Sciences, University of Oxford, Oxford, United Kingdom; ^3^ Centre for Sustainable Healthcare Education, Faculty of Medicine, University of Oslo, Oslo, Norway; ^4^ Centre for the Study of Digitalization of Public Services and Citizenship, Oslo Metropolitan University, Oslo, Norway

**Keywords:** loneliness, social isolation, technology, telepresence, policy analysis, sociotechnical, interpretive, qualitative

## Abstract

Loneliness is represented in UK policy as a public health problem with consequences in terms of individual suffering, population burden and service use. However, loneliness is historically and culturally produced; manifestations of loneliness and social isolation also require social and cultural analysis. We explored meanings of loneliness and social isolation in the UK 2020–2022 and considered what the solutions of telepresence technologies reveal about the problems they are used to address. Through qualitative methods we traced the introduction and use of two telepresence technologies and representations of these, and other technologies, in policy and UK media. Our dataset comprises interviews, fieldnotes, policy documents, grey literature and newspaper articles. We found loneliness was represented as a problem of individual human connection and of collective participation in social life, with technology understood as having the potential to enhance and inhibit connections and participation. Technologically-mediated connections were frequently perceived as inferior to in-person contact, particularly in light of the enforced social isolation of the COVID-19 pandemic. We argue that addressing loneliness requires attending to other, related, health and social problems and introducing technological solutions requires integration into the complex social and organisational dynamics that shape technology adoption. We conclude that loneliness is primarily understood as a painful lack of co-presence, no longer regarded as simply a subjective experience, but as a social and policy problem demanding resolution.

## Introduction

Loneliness has been recognised as a global public health issue, exacerbated by measures of lockdown and social distancing during the COVID-19 pandemic (1, 2). Loneliness is also perceived as an economic and political threat to society, with the potential to cause disengagement in democracy (3). Although recognised internationally as a health issue, the UK government is one of few to take significant steps to address loneliness as a policy problem. In 2018 the first “Minster for Loneliness” was appointed who launched *A connected society: a strategy for tackling loneliness – laying the foundations for change*. The strategy highlighted the adverse health consequences of loneliness, which was linked to early deaths on a par with smoking and obesity and associated with increased risk of coronary heart disease, stroke, depression, cognitive decline and Alzheimer's Disease (4).

Both loneliness and social isolation are considered to be social problems. Loneliness is generally perceived to be a painful subjective experience (such as can be felt even in the middle of a crowd) and social isolation as an objectively measurable limited social network. Loneliness leads to health problems but the causes of loneliness are linked by policy to social problems, necessitating changes such as more connected communities. Loneliness is recognised as being socially produced and historically situated (5). Technology has an important role in both enhancing and endangering the positive human connections that can reduce loneliness and social isolation. This paper contributes to the understanding of the relationship between technology and loneliness and to the emergence of loneliness as a social problem.

Theories of loneliness range from socially constituted patterns of isolation related to social identity and situation (6, 7) and biological and evolutionary constructs of humans as essentially social beings (8). Loneliness can be construed, from an existential perspective, as an essential human experience indicating the social nature of humanity (9). The importance of the interrelationship between cognitive factors and social situations in producing loneliness has emerged from psychological studies (10). Regardless of the diverse theoretical underpinnings of how loneliness is understood, *presence* is a potential solution, with telepresence made possible by technology. Such tele – or virtual – presence involves human connection at a distance, via video and audio technology. This differs from co-presence, or physical presence which is often synonymous with “real” or spatial presence: being with someone, in physical proximity, sharing time and space. This study focuses on the social meaning and use of telepresence technologies. We draw on interpretive approaches, using Bacchi's *What's the Problem Represented to Be? (WPR)* framework to analyse the policy problem of loneliness (11), and take a sociotechnical approach to considering the relationship between loneliness and technology.

In this paper, we analyse the use of telepresence technologies to argue that loneliness is understood as a problem of individual human connection and one of collective participation in social life. We found technology is understood as having the potential to facilitate human connections with positive and negative consequences. Technologically-mediated connections were frequently perceived as inferior to in-person contact, particularly in light of the enforced social isolation of the COVID-19 pandemic. We argue that addressing loneliness requires attending to other, related, health and social problems and introducing technological solutions requires integration into the complex social and organisational dynamics that shape technology adoption. We found that the work that goes into addressing loneliness includes campaigning work to identify and explain the problem.

This paper contributes to the literature on loneliness and technology by analysing the dynamics between technology, loneliness and health, bringing new insights into how loneliness is understood in contemporary UK society. The paper is organised as follows; first we provide the background to the study with an overview of literature that has examined the relationships between loneliness and technology. We then explain our methodological approach and describe our empirical data and approach to analysis. We present our findings about how the problem of loneliness is represented in our data before discussing the dualism and duality of the relationship between loneliness and technology.

### Background

The relationship between technology and loneliness is frequently represented as a dualism: the relationship has positive and negative characteristics with technology understood as having the power to both cause and cure loneliness and social isolation. Nowland et al.'s psychologically informed work captured this dualism in their representation of the relationship between social internet use and loneliness as *bidirectional* and *dynamic*; when used for social connections the internet can enhance relationships but when used to withdraw from social interaction it can exacerbate loneliness (12). The dualism of the relationship between technology and loneliness has also emerged from studies in diverse fields including medicine, public health, health services research, media and communications. Broader sociological and historical studies of loneliness provide further perspectives on this dynamic. We outline these varied perspectives before offering an approach to understanding the relationship between loneliness and technology which moves from dualism (recognising the separate but paired elements of the relationship) to duality: recognising the interdependence and mutually constitutive nature of the relationship (13, 14).

The “modern technological world” has long been feared as contributing to social isolation by engendering social changes, for example, changes to employment and family life which reduce positive human attachments (15, p. 52). The social and psychological impact of the internet has been observed as having a paradoxically negative impact on loneliness and social isolation by Kraut et al. in their 1998 survey of American households (16). Similar concerns have been investigated by Turkle about the “always on” nature of increased technological connection, and the changing nature of intimate and authentic relationships with, and mediated through, technology (17). More recent empirical research of the relationships between technology and loneliness has also found negative associations. Problematic internet use [defined as a preference for online social interaction over in-person relationships with negative social and cognitive outcomes (18)] has been found to be associated with loneliness amongst younger and older adults (19, 20). Studies reporting benefits of technology for social isolation, such as use of social media during the pandemic, also report accompanying problems such as increased anxiety (21). Fang et al, investigating whether technology might have negative consequences for older people, found it to be a “mixed blessing” with potential for poorer psychological outcomes amongst lonelier older adults (22, p. 530). Further, not feeling proficient with technology was found to increase feelings of social isolation for older people during the pandemic (23); not being technologically connected can provide a new dimension of isolation.

Despite the potential dangers technology is purported to hold, it has been frequently used as an intervention to address loneliness and the associated adverse health outcomes, particularly with older people. Technologies have been studied as ways of detecting, preventing and alleviating loneliness and social isolation with varying degrees of success and different theoretical models of how technology might work, or not.

Detection of loneliness using technology involves wearables and home sensors capturing data about the activity patterns of individuals. Data captured on mobility within and outside the home, communications, and sleep patterns have been studied in comparison with measures of loneliness and social isolation for younger (24) and older cohorts (25, 26). Although uncertainties remain as to the effectiveness of this kind of detection, not least in relation to ethical and privacy issues (27), this approach makes it possible to develop biomarkers or phenotypes of loneliness and to use machine learning to predict loneliness levels (28). Loneliness and social isolation are understood in these terms as identifiable by, and constitutive of, certain behavioural patterns.

Different types of technological interventions for older people have been evaluated as having positive effects in alleviating and preventing social isolation. These include remote provision of services [e.g., therapeutic interventions and self-guided therapy (29)] and participation in specifically designed interventions such as a virtual senior centre (30) and an Internet Information Station (31). Both mainstream [e.g., mobile phones and laptops (32)] and especially adapted technologies [for example ‘Skype on Wheels’ (33) and personalised tablets (34)] have been found to be beneficial for older people experiencing social isolation in diverse settings. Many of these interventions included support and training in using digital technologies to enable them to connect with family and friends and engage in new online activities. Wang et al. noted the importance of such training and assistance for interventions to be successfully implemented and raised the question of whether it is the technology or the support provided that offers most benefits (35).

A number of evidence reviews suggest technology can have beneficial effects on social isolation and loneliness for older people (36–41), however, effects may be short lived (42) and the causal relationship of technology on loneliness has been interpreted by Casanova et al. as weak (43). On the contrary, Shah et al's systematic review and meta-analysis found no evidence of effectiveness of digital technology interventions on loneliness in older adults (44). Other reviews expressed caution about mixed evidence of positive effects of technologies on loneliness in older adults (45) and the need to consider technology as just one intervention that could be beneficial amongst others (46, 47). Reviewing the evidence about loneliness and social isolation for older people during the pandemic led Kasar and Karaman to call for greater use of technology, whilst recognising the barriers that some older people face which might be addressed through free communication platforms or simply telephone calls to support them to be socially active (48). The evidence of whether technology *per se* might be effective in addressing social isolation and loneliness is mixed, albeit generally interpreted optimistically.

In addition to the body of evidence about older people, there are also studies of how technology can facilitate social connection for the general population in the context of the COVID-19 pandemic (49, 50) and other specific cohorts including people with mental health problems. Toh et al.'s evidence review of digital interventions for loneliness for people with mental health problems found different types of technology used as interventions for loneliness: remote (web and phone based) and blended (a combination of remote and in-person) interventions, virtual reality and socially assistive robots (51). Although there were no clear conclusions of clinical effectiveness found in the review, the potential for technology to improve social isolation for people with mental health problems was recognised. During pandemic conditions, technology was identified as being particularly important in providing social support (52) and in Gillard et al.'s study of the impact of the pandemic on people with pre-existing mental health problems, digital interfaces were able to offer continuity of care to some people from their service providers during periods of lockdown (53).

In considering how technology might alleviate loneliness, few studies provide theoretical frameworks or explanations (40). Those that do categorise technologies and offer explanatory models to account for empirical findings, drawing on theoretical frameworks that range from evolutionary to ecological approaches. Shah et al.'s scoping review produced categories of technology for older adults (social networks, messaging services, video chat, virtual spaces or classrooms with messaging capabilities, robotics, games, and content creation and management) and different purposes for those technologies (social communication, social participation, a sense of belonging, companionship, and feelings of being seen and different) (54). A model of how assistive technology mitigates social isolation and loneliness in older people was developed by Jutai and Tuazon which linked the steps of accessing health care, care/support seeking behaviours, increased resilience (through technologies such as games that encourage cognitive function) and social support through technologies that enable people to connect (55). This reflects an ecological framework which considers isolation as an outcome of the interaction between individual, relationship, community, and societal factors (36). In contrast, Masi et al. noted the importance of correcting maladaptive social cognition (comprising negative responses to social connections which make it harder to connect) to reduce loneliness, rather than simply addressing social skills, social support, or opportunities for social interaction through technologies (56). There is a divergence between evolutionary and ecological frameworks which represents differences between social/situational and biological (including physiological and psychological) theories of loneliness, with corresponding variations in how technology is understood to help. Using technology to leverage community resources, strengthen networks (34) and engage with the outside world (57, 58) offer routes to addressing social isolation and situated loneliness (59). Alternatively, technologies can address the “regulatory loop” of internal thoughts and feelings which protects people from negative social contact but ultimately reduces positive connections (56).

Two important issues arise when considering the limitations of technology, or how it might *not* work to address loneliness: comparisons between technological and human connections and problems of adoption or implementation of technology. Technology-mediated communication compared unfavourably with the “gold standard” of in-person presence in Burholt et al.'s study of family contacts with older people; telephone, text and video contacts were found to be neither functionally nor emotionally equivalent to embodied, physical co-presence (60, p.1209). Technology is reported as being unable to replace face-to-face social interaction (61), and maintaining therapeutic relationships via digital interfaces can be difficult (53). Technology designers have responded to the challenges of producing more satisfactory experiences of technology-mediated communication – as shown in increased provision of video-calling during the pandemic compared to audio-only telephone contact – and through embodied technologies such as the therapeutic social robot, Paro, and HUG, a therapeutic calming device for people with dementia (62). Paro and other robot companions offer embodied co-presence which in itself can provide company thus addressing loneliness, but also stimulate social connections with other people (37, 63).

More prosaically, but of critical importance, technology fails to address loneliness and social isolation when it does not work due to poor connectivity or it is not available when people do not own devices or have the skills, knowledge or dexterity to use technology, as highlighted by Adedeji et al.'s scoping review of technology and social isolation and loneliness amongst older people in African countries during the pandemic. Ineffectiveness in that context was associated, in part, with poor infrastructure and the digital divide (lack of internet access, devices, and disposition towards technology (61). Usability, acceptability and availability are necessary for technologies to be adopted to address loneliness, as is technical competence, with additional difficulties apparent for older adults as well as people digitally “excluded” (64).

The dualism of the relationship between technology and loneliness permeates the research evidence, and is relevant to various conceptual models of how loneliness occurs and therefore how it might be caused and alleviated. Technology can mediate human connections with the “external” world and improve the “internal” world of emotion and cognition (57). Despite a growing body of research that considers if and how technology might alleviate loneliness and social isolation, particularly for older people, there is little known about how technology and loneliness are interrelated to broader social, policy or historical perspectives and how technological solutions to loneliness are interpreted in practice. This paper will contribute knowledge of how loneliness is perceived and represented in relation to the emerging phenomenon of telepresence, drawing on empirical research of these technologies in use.

## Materials and methods

### Methodology

Our overall approach for this study was to analyse the dialectics of loneliness and technology. By studying the technologies designed to address loneliness, we sought to understand the contemporary meaning of loneliness in the UK and, reciprocally, how technologies were shaped by understandings of loneliness. This methodology owes much to Bacchi's *What's the Problem Represented to be (WPR)?* approach (11) which starts by examining a policy solution in order to better understand the nature of the problem that the solution is intended to address (65). Bacchi approaches policy as discourse (66), rejecting the idea that problems such as loneliness are simply naturally occurring and need to be solved, but instead that they are constituted in certain ways in the discourse. This is not to suggest that loneliness and other problems are not real, but rather that the ways in which those problems are framed and described deserves to be analysed, not least because the framing of a problem generates certain solutions and negates others.

We studied telepresence solutions to loneliness through practices as well as through policies. We drew on a sociotechnical framework [Non-adoption, Abandonment, and Challenges to the Scale-Up, Spread, and Sustainability of Health and Care Technologies or NASSS (67)] to guide our study of the adoption of loneliness technologies. A sociotechnical approach to investigating technologies takes account of the interactions between people and technology and is concerned with how technologies in use are interpreted, and how their use shapes and changes the activities of the people who use them. The NASSS framework considers the adoption (or non-adoption) of technologies in relation to the longitudinal dynamics between a technology, the conditions a technology is intended to address, adopters, organisations involved in implementing the technology and the wider policy and economic environment. We therefore extended Bacchi's approach to considering not only how the problem of loneliness and the solution of technology was represented in policy, but how it was represented in practices of adoption of those technologies.

**Figure 1:**
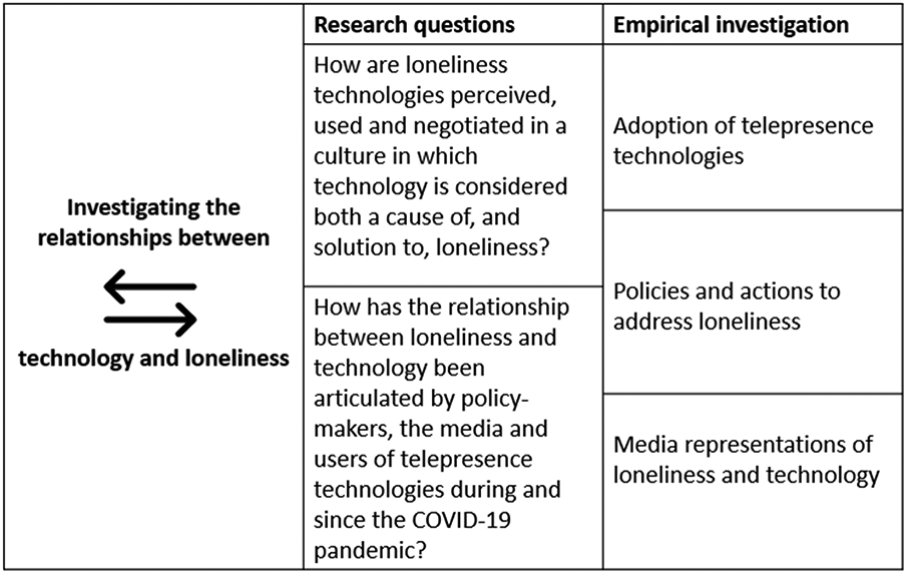
Overview of study design.

Our methodological approach was intended to “unpack” the cultural dialectic between loneliness and technology, and in particular to ask the research questions: How are loneliness technologies perceived, used and negotiated in a culture in which technology is considered both a cause of, and solution to, loneliness? How has the relationship between loneliness and technology been articulated by policy-makers, the media and users of telepresence technologies during and since the COVID-19 pandemic? We provide a diagrammatic overview of our study design in Figure 1.

### Research setting

We studied the relationship between loneliness and technology in the UK by following the adoption of two technologies (KOMP and AV1) from 2020 to 2022 as part of a wider study of telepresence technologies (65). We also studied the broader social and policy context for the adoption of these technologies in the UK by tracing efforts to address loneliness (by politicians, policy-makers, campaigners and service providers) and media representations of loneliness and technology.

The two telepresence technologies selected for this study were developed by a Norwegian start-up company, No Isolation, specifically to address social isolation and loneliness. The company was founded in Norway in 2015 and their UK office opened in 2017. No Isolation has a mission to:

“…reduce involuntary loneliness and social isolation by developing communication tools that help those affected” (68).

AV1 is a distance learning avatar. Inspired by the experiences of a young person with cancer who could not attend school, AV1 is a small robot-like device that physically represents the young person or child in their classroom. The young person or child connects to the AV1 from home (or hospital) via an app on their smartphone or tablet. The young person or child can see and hear the activities of the classroom and interact with their classmates and teacher through the avatar. The student at home is not visible from the classroom. KOMP is a “one-button”, large-screen computer designed for older people who are not comfortable with or able to use devices such as touchscreens. KOMP, which resembles a television, connects from the older person's home to family members via their smartphones or tablets. KOMP has been deliberately designed to have limited functions so it is easy for people to use and so can receive but not transmit video calls, photos and text messages. Although originally designed for individual family use, a KOMP Pro version has been created by No Isolation to be used by service providers to connect with older people they are supporting, for example in care homes.

Both devices are described as telepresence technologies, that is they offer presence at a distance to combat loneliness and isolation. KOMP and AV1 were introduced into the UK at a time of increased policy focus on the problem of loneliness. The first government strategy to tackle loneliness was launched in 2018 by Tracey Crouch MP, appointed as the first Ministerial lead for loneliness (4). The strategy was dedicated in the House of Commons by Tracey Crouch to the memory of Jo Cox, the MP murdered in her consistency in 2016 and who had campaigned against loneliness during her career, including through the establishment of a cross-party commission of MPs and charities (69, 70).

Technology featured in the first government strategy against loneliness as both threat and opportunity. In the strategy, technology is linked with societal changes that reduce opportunities for human interaction. Further, the potential for online harms for children and young people are noted. However, this strategy set out the government's commitment (via the Department of Digital, Culture, Media and Sport) to harness the power of technology to increase social connections and address loneliness, and featured AV1 as an example of the positive role that technology can play in addressing loneliness amongst children and young people (4, p.45). AV1 received further policy support from the Department of Education in 2018 through the Alternative Provision Innovation Fund which supported the trial of AV1 to assess telepresence to support children and young people to access education whilst unwell (71, 72). By 2020, No Isolation reported working with more than 20 local authorities across England where AV1 was used by more than 400 children or young people, the number of local authorities had risen to 50 by 2023 (*data from meeting observation October 2020*) (68). Charities also purchased AV1s to support children and young people in line with specific causes, for example childhood cancer.

Technology was considered to have potential to support older people with loneliness by facilitating ongoing relationships as well as being the catalyst for new social connections. Yet inaccessible technology for older people was concerning as it could potentially create barriers to social connection (73). KOMP's design aimed to remove any such barriers of accessibility for older people through its careful design to make use easy. By 2020, KOMP was in use in Norway by more than 500 people with around 80 devices in use or being trialed in the UK (*data from meeting observation October, 2020*).

By April 2020 the UK was in lockdown due to the COVID-19 pandemic. Policy attention turned again to loneliness with a specific government plan to tackle loneliness and social isolation during this period which included a public campaign (#Let'sTalkLoneliness), a Tackling Loneliness Network and funding for charities [e.g., (74)]. Technology was widely used to connect people during lockdown and consequently the need to support people with less access to technology became a policy issue (75).

### Methods and data

Studying the adoption and use of AV1 and KOMP before and during the COVID-19 pandemic gave us the opportunity to analyse how loneliness and social isolation were understood as problems that required solutions. We analysed the practices of spread (e.g., from Norway to UK), scale-up (from pilot projects to having greater impact), adaptation over time (as the technology was introduced into different settings and in the varying contexts of pandemic social distancing and post-lockdown life) and sustainability (becoming embedded in service provision) to find how funding and use of the technologies were justified and evaluated by a range of organisations.

We studied the adoption of KOMP and AV1 and the broader context for the adoption of these specific technologies to address loneliness. We collected a range of data: interviews, documents (including policy documents, local evaluations and media articles) and fieldnotes from observations. Participants were people actively working to adopt KOMP and AV1 and other stakeholders in loneliness policy. In 2020 we interviewed 20 people directly involved in adopting AV1 and KOMP (including teachers, local authority education specialists, social services managers) and conducted 8 follow-up interviews between 12 and 13 months later to trace how the technologies had been adopted over time. We also interviewed 10 stakeholders who were involved in addressing loneliness as a campaigning and a policy issue. We observed 17 meetings relating to loneliness and technology between 2020 and 2022, including meetings to discuss KOMP and AV1 and wider public meetings relating to loneliness campaigns. We reviewed eight debates about loneliness conducted in the House of Commons and one in the House of Lords between 2018 and 2023. We selected 140 media articles that included mentions of loneliness and technology. See Table 1 for a summary of data sources.

**Table 1:** Summary of data sources.

Data types	Participants/events	Data items
Adoption of telepresence technologies		
Interviews concerning adoption of AV1	Nine interviewees from 2 × mainstream schools (1 × primary and 1 × secondary), 2 × specialist education providers (1 × hospital school, 1 × alternative provision) 1 × education advisor, 2 × charities, 2 × local authorities	9 interviews + 4 follow-up interviews = 13 interviews
Interviews concerning adoption of KOMP	Eleven interviewees: 3 × charities, 6 × local authorities, 2 × NHS	9 interviews (2 interviews were conducted in pairs) and 4 follow-up interviews = 13 interviews
Meetings about technologies	Discussions with technology developer, observations of discussions between technology developer and potential adopters, discussions of evaluations	14 meetings
Documents about technologies	Documents produced by adopters to support implementation and evaluate technologies	19 documents
Policies and actions to address loneliness		
Stakeholder interviews	4 from charities, 1 from business, 1 politician, 1 campaigner, 2 from think tanks, 1 independent consultant	10 interviews
Documents from stakeholder interviews		15 sources (including reports, podcasts, short film)
Events	Loneliness week events, report launch event	3 events observed
Documents from events		3 documents
Policy documents	National and local government strategies and annual reports	12 documents
Political debates	House of Commons debates (8)	8 debates recorded in Hansard
	House of Lords debate (1)	1 debate recorded in Hansard
Media representations of technology and loneliness		
Media articles	Media stories pre and post COVID-19 pandemic	141 newspaper articles

Participants involved in adopting the devices were initially recruited to the study via No Isolation, who sent an invitation on behalf of the research team to 13 people involved in organisations that had adopted either AV1 or KOMP. Participants were asked to respond directly to the research team, and all those who responded consented to be interviewed, with a further seven being identified through snowballing (suggested by the interviewees as potential participants). Stakeholder interviewees were identified through discussion with No Isolation, from observations of events, policy documents and campaigns and through further snowballing. Nineteen stakeholders were approached and ten agreed to be interviewed. Very few politicians consented to be interviewed, which led us to search parliamentary debates for discussions of loneliness, nine such debates were identified between 2018 and 2023. All participants gave informed consent. Ethical approval was provided by the University of Oxford Interdivisional Research Ethics Committee (9 February 2021 reference number R73899/RE001).

Interviews and observations were conducted online by one of 2 authors (GH or LM) via MS Teams or Zoom. All but one interview was audio-recorded and transcribed. Contemporaneous notes were made for the interview that was not recorded (due to technology failure). Interviews lasted between 32 and 64 min, resulting in a total of 1,144 min of audio data. Sources which were referred to during interviews (such as policy documents, briefings, evaluations, podcasts, publications) were collated by the research team for analysis. We reviewed 49 sources including documents concerned with evaluating and disseminating KOMP and AV1, loneliness campaigning materials and policy documents. Meetings suitable for observation were identified in a similar way, with initial meetings being suggested by No Isolation which included developer team discussions about new projects and pitches to prospective adopters. Other events were identified through snowballing, interviews and networking including a range of activities that took place as part of national loneliness week in June 2022. Meetings were observed by 1 of 2 researchers (GH and LM) who made contemporaneous notes and collated documents identified for analysis.

We also set out to understand the broader representation of technology and loneliness through an analysis of media articles. Using a similar method to Mroz et al. we developed a search strategy to find media articles about loneliness and technology on LexisNexis Academic UK (76). We selected two periods: 1 December 2017–31 December 2018 which was a significant period in the development of loneliness policies in the UK with the Jo Cox Commission for Loneliness report being finalised on 15 December 2017 and the UK government's loneliness policy launched in October 2018. The second period was 16 March 2020–23 July 2021, from the official start of lockdown in the UK to so-called “freedom day” when most legal limits on social contact were removed (77). Our search terms were: “loneliness” OR “social isolation” AND “technology”. Live updates, Twitter feeds and duplicate articles were excluded, resulting in a total of 1,101 articles (282 from period 1 and 819 from period (1) We then sampled every seventh article from the results to create our dataset of 140 articles (37 from period 1 and 103 from period (2). The dataset of 140 articles were from 10 national newspapers (see Table 2) and included news articles, features on health, technology, business, as well as lifestyle and commentary pieces.

**Table 2:** Summary of newspaper articles.

Publication	Number of articles included pre-COVID-19	Number of articles included post-COVID-19
The Daily Mail and Mail on Sunday	2	4
The Daily Mirror and Sunday Mirror	0	5
The Daily Telegraph	14	38
The Express	2	2
The Guardian	8	18
The Independent	3	15
The Sun	1	5
The Sunday Times	1	4
The Sunday Express	1	1
The Times	5	11
Total	37	103

The total dataset comprises 251 data items (36 interviews with 30 participants, 17 observations of meetings or events, 58 sources reviewed including policy documents and debates and 140 media articles).

Analysis of the dataset involved two distinct stages. We initially grouped the data into sub-categories: (1) adoption of telepresence technologies, (2) broader policies and actions to address loneliness, and (3) media representations of loneliness and technology (see summary Table 1 for further details of the dataset). The first stage of analysis focused on the data in each sub-category, followed by a thematic synthesis across the dataset. Analysis of adoption of telepresence technologies was conducted by LM and GH who started with narrative analysis (78) of the interview data to trace the practices of adoption of the technologies (including how the technology was first introduced, how it was funded and supported, and, where possible, how use had changed over time) and thematic analysis to identify how loneliness was articulated and how the technologies were understood to address loneliness. Analysis of policies and action to address loneliness was conducted in a similar way by LM and GH. Analysis of media articles was conducted by MH and GH, with EEJ and TS. Two researchers (MH and GH) read all the articles in the dataset. MH created a series of inductive codes which were discussed with other authors (GH, EEJ and TS) and refined to create broader themes. GH and LM then considered the commonalities across the three sub-sets of data in dialogue with Bacchi's WPR questions, asking of these themes: what's the problem of loneliness represented to be? What assumptions underpin this representation? How has this representation come about? What is left unproblematic or “silent” in this representation? What are the effects of this representation of the problem? How has this representation been dissemination and how could it be challenged or disrupted? Our results are reported below.

## Findings

Loneliness was commonly represented in UK policy as a public health problem; causing ill health to a similar extent as other major public health problems such as smoking and obesity (3, 68). Despite the personal nature of loneliness acknowledged in the first loneliness strategy, a case was made for government intervention to prevent people feeling lonely. The need for intervention was linked in large part to the representation of loneliness as a health problem with consequences for costs to the NHS and the potential burden on services. Loneliness was also represented as a social problem; an example of social injustice that is damaging to humanity (4, p.2). Our analysis of how the relationship between loneliness and technology was articulated in policy documents, and in the practices of addressing loneliness, shows that loneliness was represented variously as a problem of individual connections, of participation in the norms of social life and as a campaigning social issue. The urgency of the “crisis” of loneliness (*as described in an interview with a senior charity officer*) and the moral imperative to address this crisis was emphasised by stakeholders campaigning to address loneliness, as one said:

“…it's pretty horrific to deprive people of something that's fundamentally critical to our humanity…” (*interview with independent charity and policy consultant*).

The limits of technology in addressing loneliness and associated problems were also discussed by our participants. The campaigning and influencing work apparent in our data reflected broader policy and social concerns about the nature of a successful society. We consistently found loneliness represented in our data as a serious problem to be solved, or cured, not one that might be tolerated, endured or to have any beneficial aspects. Following Bacchi's approach of considering what is *not* said or is silent in our data, we found few neutral or positive representations of loneliness other than descriptions of the pain of loneliness compared to peaceful solitude (*from interview with experienced loneliness campaigner*).

### Individual connections

The importance of fostering connections between individuals permeated our data and underpinned much of the work that our participants engaged in around developing and adopting technologies and promoting loneliness policies and campaigns. The technologies we studied were designed specifically to make connections between people unable to be together in person due to distance caused by the social isolation of old age (KOMP) or that imposed by long-term illness (AV1). The COVID-19 pandemic caused new concerns about a wider range of people likely to suffer from loneliness and social isolation in lockdown, and caused greater efforts to provide people with a range of technological devices, skills and support to facilitate connections, including funds made available by the government, e.g., (74). KOMP, designed to address loneliness through personal connections, was also put to use in connecting people with health and other services made inaccessible by pandemic restrictions. Representations of telepresence connections in general were compared unfavourably at times with in-person connections and were sometimes valued as temporary substitutes for or pathways to “real” connections as suggested in an article advising on how to make friends as an adult:

“Use tech to make connection then take it offline” (79).

Not all telepresence connections were straightforward to achieve, and some led to negative rather than positive experiences.

The original intention of KOMP, to connect an older person with family members, represented a certain “script”, which informed the design of the so-called “warm” technology by No Isolation to mediate positive family relationships (80). The power of technology to foster beneficial connections was frequently represented in our data, particularly during conditions of lockdown. Jamie Stone MP's contribution to the House of Commons debate on COVID-19 and loneliness included the story of Sally, one of his constituents who had found some relief from loneliness through technology:

“…she told me that one of her grandchildren had zoomed in and, for all the difficulties of this way of talking to each other through a small screen, the grandchild saying, “Hello, Granny. How are you?”, really gave a little lift to her day…” (75).

Human to human connections were the primary focus of our data, but AV1 was also reported as being used to allow a child missing their pet to connect from a distance during a long-stay in hospital *(interview with an alternative education provider)*. However, not all such technology-enabled connections were represented positively in our data. Connections via KOMP could be experienced as intrusive (*interviews with local authority providers and charity officers*) and concerns about AV1 “spying” on teachers and students were found in our data (*interviews with primary school teacher and council officer*), reflecting privacy concerns reported elsewhere (81). Connections which were already fraught could be continued through technology; one participant (*alternative education provider*) explained how the bullying of a child by their classmates was perpetuated via AV1. Concerns about the power of technological connections to cause harm, and indeed to contribute to loneliness, were commonly represented in media articles in fears that included the addictive and negative habits of “zombie scrolling” (82), inauthentic online friendships (83), cyber bullying (84) and abuse (85).

“Off-script” connections were established through KOMP and AV1 as the technologies were adopted in different settings than originally envisaged; telepresence was used to connect people to address issues other than loneliness including service provision during the pandemic and to mediate other connections where in-person connections were considered risky. For example, a healthcare provider trialed the use of KOMP during the pandemic across multiple service settings to conduct assessments and provide therapies whilst avoiding in-person contact and therefore potential COVID-19 transmission both in patients' homes and within clinical settings. In this way, KOMP was used like other telemedicine technologies during the pandemic as a kind of “digital PPE” (86). Providing a protective connection at a safe distance was a function of AV1 in one reported example of re-integrating an excluded child, first back into the school and then eventually into their classroom (*from interview with education officer at local authority*). The risk in this example was not COVID-19 infection but the safety of the child and their classmates. In these cases, the solution of technology-mediated connections was targeted at problems other than loneliness.

We found the use of telepresence technologies to connect individuals emphasised the importance of those connections, whilst the limitations and troubles of certain connections indicated different causes of difficulties in connecting. Difficulties in connection included the common problems of social distancing measures during the pandemic and concerns about safe, meaningful, beneficial human connection.

### Participation

Telepresence technologies facilitated social, collective connections which enabled people to participate in a range of activities at a distance including school, work, and leisure activities. A feature of our data on the use of KOMP and AV1 was the ability for users to participate in social life. Important prior to the pandemic, telepresence became widely used to enable people to share in social events at a distance, such as the KOMP user described as:

“… able to share his sister's birthday who he hadn’t seen for a long time…he was there when she was opening her presents and [….] her cake” (*interview with health service provider)*.

Participating in social events using telepresence technologies shifted the focus from individual connections, for example between grandparent and grandchild, to connections between groups including families and classes. One participant reported the benefits of KOMP for one older person unable to visit their younger family members in terms of allowing them to be:

“…part of the family again because they’re in their houses as well and they’re seeing the kids paint and cook or, you know, sing songs to them. It's being a part of that again” (*interview with charity officer)*.

Connections between social groups were implicated in the use of AV1 as the technology provided a telepresence connection between student and class, but also mediated a social relationship between family and school. Families were involved in supporting the technology required for the telepresence connection and, through social agreements about the use of AV1, in allaying fears of unwarranted surveillance and recording of classroom activities. The connection that AV1 offered was therefore *collective*. Conceived as a device to help a child or young people to maintain social contact during long-term absence from school due to serious health conditions, AV1 was understood by those involved in adopting it as a way for young people to participate in social life beyond the home and family including engaging in formal education and participating in the life stages of childhood and adolescence and the associated transitions. In comparison, KOMP was unable to effectively connect groups; it was unable to facilitate or replace in-person groups during pandemic restrictions. Instead, platforms such as Zoom were more commonly used for “scalable sociability” (87).

The Alternative Provision Innovation Fund launched in 2018 supported the trial of AV1 in multiple schools with the aim of improving educational outcomes for children and young people unable to attend school for a range of reasons including illness and exclusion (72). Whilst the focus of the innovation fund was on educational outcomes, the importance of participation in social life was emphasised by our participants. People involved in adopting or supporting the adoption of AV1 reported how the telepresence technology enabled children to participate not only in education, but in the social life of schools. The technology was initially regarded by many as a novelty but it was the normality of interaction and participation that was valued; chatting at playtime was as important as asking questions in class, as reported by one primary school teacher:

“They love spinning them all the way round to see what's going on … and because the head lights up when you want to answer a question it means you can participate in the class discussions” (*interview with primary school teacher*).

Participating in “normal” learning and normal life was regarded as an important benefit of AV1, when compared both with other options for providing education to children and in comparison with the unusual events that had caused them to be isolated at home. An Alternative Provision educator reflected on the benefits of AV1 over the provision of medical tuition which would otherwise be offered to children unable to attend school:

“I think, I mean the major advantage in my mind is that it enables that every young person to carry on with their normal learning. So from, with their normal members of staff sat in the class with their, even though it's remotely, but with their peers and their friends [um] during the normal, the learning that they’re doing. So, you know, like I said with medical tuition you’re relying on somebody coming in and being able to pick up what you were doing and you’re also relying on schools remembering to send work home and all of that. But with the AV1, you know, that little bit is life as normal” (*interview with alternative education provider)*.

Participating in ordinary life was valued more than any specific educational benefit, as one educator reported about a child with cancer:

“…his mum would say it was one hour of pure joy, they couldn’t see him they couldn’t see that he was swollen from his steroids, his liver had failed, he was an ordinary boy in an ordinary class and, and she said it was like one hour of pure joy and just a reminder of everything, it didn’t make him feel envious he was just glad to be back with everybody in his class” (*interview with alternative education provider*).

AV1 was not widely used during the pandemic, when schools were mainly closed and all students participated in remote learning, typically using Google classrooms or holding lessons via Zoom.

Participants working with AV1 identified the interconnections between social support, recovery from illness and participating in education. Maintaining connections and participating in social life over time was understood as being especially important for children and young people undergoing the developmental stages of childhood and adolescence, physically growing and changing and participating in different stages of educational settings. AV1 was used to enable students to participate in developmental transitions whilst physically distant from their peers, for example in the move from primary school (year 6, aged up to 11) and secondary school (year 7) as described below:

“… we’ve had children where it's spanned their transition to secondary school, they’ve started in year 6, they’ve not seen their, their peers in year 6 and then when they go back to school they’re starting a new school. But we’ve used an AV1 in that instance to do a tour of their new school” (*interview with education specialist at local authority*).

AV1 was also used to support transitions back into school for children who had been absent for reasons other than long term medical problems, such as school “refusers” and those who needed additional support integrating back into school after being isolated during the pandemic. The importance to children unable to attend school of not “being forgotten” was underlined in several interviews. Not being forgotten by others was also an important benefit reported by adopters of KOMP.

The collective nature of the connections that were made possible by telepresence were also apparent when considering the responses to AV1 by the children and young people in the schools and classrooms. Children and young people at school were involved in “looking after” the AV1, offering care to the device and to their remote classmate. There were advantages reported for those classmates as well as for the primary user as they were able to maintain relationships with their absent friends. One participant felt that interacting with and via AV1 promoted kindness and caring amongst the class, and contributed to new experiences and relationships:

“AV1 […] allow for new relationships to develop through as you say through carrying the robot around and kind of creating new experiences rather than just passively experiencing the same experiences…” (*interview with primary school teacher*).

The benefits (and limits) of technology in allowing people to participate in social activities including work and leisure were commented on extensively in our media data. The potential advantages of collective connections through platforms that offered virtual spaces for collective social activities (e.g., Gather) were compared with the intensity of one-to-one connections (e.g., via Zoom) (88). Despite the possibilities for collective telepresence experiences offered by a range of software, such as watching films and listening to music in remote groups, fears about the “dystopian” possibilities of a more permanent move to online life were common (89). Virtual participation was understood as a transitional or temporary benefit until “normal” social life might resume.

In sum, telepresence offered a way of sustaining connections and enabling participation in “normal” social life which were understood to have multiple, interrelated advantages, of benefit in circumstances which were exceptional (such as the pandemic) and unusual (for children and young people unable to attend school) as well during the isolation associated with old age.

### The limits of technology

Across our data, we found representations of the benefits of technology enabling connection and participation interwoven with acknowledgements of the limitations of technology. Telepresence was frequently compared with in-person or face-to-face presence and the full range of human connections, including touch, were noticeable by their loss. Technical “glitches” also impeded participation.

Connecting via technology was not always an entirely satisfactory experience. The “small screen” referred to in the House of Commons debate above was one example of reduced sensory input. Technology-mediated connections could not replicate human touch for people suffering from the isolation of pandemic-induced lockdown to the extent of reported ‘touch deprivation’ or ‘skin hunger’ (90). The limitations of telepresence and the need for human in-person contact was reported in the media as due to psychological effects:

“…the brain needs other humans to feel calm but it's their touch, scent and voice that has this effect, not looking at a picture of them.” (91).

Limits to what could be achieved by way of sensory connections were overlaid with concerns reported in the media about the inauthenticity of “online” relationships (92), in addition to those relationships that were felt to cause harm (93).

Technology was represented in newspaper articles and in policy documents as reducing opportunities for social contact, even whilst making certain activities easier. For example, online shopping might be convenient but prevents people from meeting in high streets and libraries (91, 94). The foreword to the 2018 government strategy points out that due to:

“…new ways of connecting and communicating with others … it's now possible to spend a day working, shopping, travelling, interacting with business and with public services, without speaking to another human being” (4, p.3).

Concerns about technology reducing the opportunity for participating in more informal social interactions, the lack of “loose cliques” (95) were heightened by experiences of the pandemic which not only prevented people seeing family and friends but involved the loss of more casual relationships:

“We are isolated from colleagues, forbidden to mix meaningfully with friends and family, and unable even to enjoy the casual pleasure of being in busy, buzzy places. Even an unmasked smile from a stranger is a lost joy.” (96).

Related concerns about the potential adverse consequences of telepresence technologies were raised by adopters of KOMP who, in recognising the benefits of social connection for older people without needing to leave home, noticed the potential for people to lose their daily living skills if they no longer had to travel to meet people (*interview with local authority officer*).

Inherent limitations of telepresence technologies in facilitating full or “real” connections were further exacerbated in some cases in our data by technological “glitches” and in their inherent limitations. Difficulties in sustaining an internet connection caused faltering use of AV1 for one of the secondary schools represented in our interview data. The school site was large with multiple wi-fi points and the AV1 moved from classroom to classroom for different lessons – unlike the usual practice in primary schools in the UK where children remain with their same teacher in the same classroom for most of their school day. The complexity of setting up and testing each wi-fi point in this case led to delays or loss of connection which, for some students, caused them to lose interest and for some teachers became too onerous. Whereas other technological obstacles to use of AV1, such as remembering to charge the device and activate each day, were more readily overcome, connecting to the infrastructure of schools was much more challenging. Firewalls in place to protect the IT systems of schools made connecting AV1 technically difficult, and sometimes reinforced concerns about security and safety (*interview with charity officer*). Difficulties in connecting novel devices to institutional IT systems were also apparent for one user of KOMP. Happily installed at home, when KOMP was taken with one user into respite care for a short period, there were difficulties in connecting to the institutional network, despite the possibility of installing roaming data in the device (*interview with local authority officer*). The individual connections that KOMP was designed to facilitate between an older person and a family network did not integrate easily with institutional care.

Our data showed that the shortcomings of technologies in providing satisfactory and authentic connections were entangled with technical failures linked to problems with accessing the internet, indicating difficulties in connecting to the IT infrastructure especially in institutional settings. These shortcomings were also entangled with fears about the role of technology in reducing social connections, with virtual presence frequently represented as inferior to in-person presence. In contrast, the importance of the reliable and familiar technology of the telephone as a way of staving off loneliness was recognised in policy and debates (73, 75, 97).

### Loneliness doesn't come alone

Loneliness and social isolation were not the only concerns of the participants of this study and were not the only problems represented in our data. We found that representations of loneliness in policy and campaigns were associated with other health and social problems. Attempts to address loneliness and social isolation through technology were inextricably linked with the situations in which people were experiencing loneliness, and other associated problems, and were affected by challenges relating to the deployment or adoption of technologies.

Loneliness and social isolation were represented in policies and campaigns as being experiences that could affect anyone, whilst acknowledging there are certain situations for certain people that can contribute to loneliness as described by Steve Reed MP:

“Loneliness affects people of all ages: disabled people who are unable to get out of the house; older people who lose friends, become housebound, and feel they lack purpose in their lives; young people moving away for work or education; teenagers coping with the challenges of growing up; and people who lose their jobs. It can affect any of us and all of us, and it can have a devastating effect on people's mental and physical health.” (69).

Identifying loneliness as something that can affect “any of us and all of us” was intended to go some way towards addressing “stigma and shame” (69), with the commonplace experiences of lockdown during the pandemic reinforcing the potential for universal experiences of loneliness. Yet some people are more likely to experience loneliness than others, and to have greater adverse effects. Work commissioned by the Deputy Mayor of London reconceptualised severe loneliness as being unequally distributed, distributed not across the life stages or ages as represented in the House of Commons debate above but associated in London with acute poverty, being single or living alone, being Deaf and disabled, going through life changes or being new to London and feeling different or experiencing prejudice (98).

The challenges of addressing loneliness in combination with other social problems were highlighted by specific events in Loneliness Awareness Week 2022 such as *Tackling Loneliness at its Roots: how can we best support low-income households to overcome social isolation?* and the APPG meeting on *Tackling Loneliness and Connected Communities: Exploring loneliness as a determinant of health and the role social prescribing can play* (*meeting held 15 June 2022*). Discussions during these meetings showed a recognition of the need for increased social and digital infrastructure to address loneliness and the lack of basis resources, including food and travel, for some people.

Participants in our study working to introduce telepresence technologies were aware that loneliness was not necessarily the only problem that needed addressing in order for their work to be successful. Those working with KOMP and older people were also concerned to address related problems of health, mobility and independence which could lead to and be exacerbated by feelings of loneliness and social isolation. People working with AV1 in educational settings recognised the importance of addressing social isolation in terms of longer-term consequences and outcomes for young people such as employment and training as well as mental health problems and their ability to form meaningful relationships. These holistic interpretations of the need to address loneliness motivated use of telepresence technologies and led to their adaptation for new purposes: AV1 worked to reintegrate a child into school safely from a troubled family (*interview with education specialist at local authority*), KOMP provided visual entertainment for an older person with cognitive decline no longer able to enjoy television shows (*interview with local authority officer*).

The technologies we studied were deliberately designed for ease of use to overcome potential barriers such as lack of digital skills or unfamiliarity with touchscreen technology. Roaming mobile data connections could circumvent problems with internet access that indicated the digital disparities for some users. Introducing telepresence technologies highlighted the complexity of social situations that could impede effective adoption, and which contributed to the need for the technology. The use of AV1 for a child in a vulnerable family implicated in antisocial behaviour, drug-taking and violence supported reintegration into school, but required negotiation. Technology could address loneliness, and other social problems, but the use of technology revealed further disparities. The relative importance of loneliness as a problem to be tackled was shown in comparison with other problems (including acute poverty) as technological solutions were considered.

### Campaigning for loneliness

There was a significant amount of campaigning and influencing work carried out by participants in this study and represented in the documents and policies analysed. Campaigning and influencing work focused on both establishing loneliness as a policy concern and in addressing loneliness. As technological solutions for loneliness were introduced, other campaigning and influencing work became necessary to address digital disparities and to promote the relevance and benefits of the proposed solutions.

Campaigning for loneliness to be taken seriously as a public policy had been a large part of Jo Cox's work as an MP, and has been recognised as an important part of her legacy. Such campaigns led to public policy responses both at national and regional level, with the role of technology recognised as an important contributing factor in addressing loneliness, along with cautions about the contribution that technology can make to exacerbating loneliness. Campaigns have also been an instrument of public policy to address loneliness, reliant in many cases on the use of technology, for example the digital platform created by the government in 2021: The Tackling Loneliness Hub (99) and the #LetsTalkLoneliness campaign (100).

Relationships between technology, loneliness and campaigning work were apparent in our data in efforts to draw attention to and address digital disparities between people with ready access to devices and the internet along with the skills required to use them and those digitally excluded. The increase of the use of technology for social connection experienced during the pandemic was widely reported in the media as was the vulnerability of certain groups of people less digitally connected. The provision of mobile phone and data packages to homeless people post-pandemic had become part of the charitable offer previously confined to shelter and food (101). Families reported as struggling during lockdown were able to connected via online games (102) and charities demanded internet access for older people as a “lifeline” (88). Actions to address digital inclusion were recommended by the Tackling Loneliness Network including improving access to devices and data (99).

Following public policy recognition of the problem of loneliness, further work was still required to influence potential adopters of technologies designed to address loneliness. No Isolation, developers of the telepresence technologies studied as part of this research, undertook work to raise awareness of loneliness as part of their mission, compiling research about the extent of loneliness and making it available to policy-makers and to potential users of their device. Their website includes a page: “learn about loneliness” and they reported needing to emphasise the potentially large number of people experiencing loneliness, particularly school students, when discussing the relevance of their technologies (*interview with team member from No Isolation*).

Adopters of AV1, having been convinced of its potential benefits for students, spoke of having to “drum up” support for the device with colleagues (*interview with education provider in hospital school*). Considerable work was undertaken to provide briefings and address concerns about privacy, security and anxieties about the potential for additional work for teachers (*interviews with alternative education provider and education specialist at local authority*). Our participants aligned the benefits of the device with the outcomes they knew were important to teachers and in education policy; identifying how AV1 might contribute towards achieving higher educational attainment and attendance for students (*interview with alternative provision provider*)*.* The relative cost of AV1 to other forms of alternative provision that would be required for children unable to attend school, such as medical tuition, was mobilised as an argument to adopt AV1, as was the lower burden of work for teachers to include the device in their classroom rather than having to prepare special work for a student to do at home (*interviews with alternative provision provider and local authority education specialist*). Similar logics were used by adopters of KOMP when considering the cost of the device, it was far more efficient to connect via KOMP than physically travel to visit an older person at home (*interview with local authority officer*).

The need to pay attention to loneliness as a policy issue and to address loneliness as a social problem was a campaigning issue involving charities, policy-makers, technology developers and adopters in mobilising different arguments about causation, consequences and benefits of technology.

### Connected societies

Representations of loneliness and technology were associated, in our data, with wider concerns about societal problems. Concerns expressed by campaigners, policy-makers, in the media and by adopters of telepresence technologies included the changes to society created by technology and the consequences of addressing loneliness through technological means. The limits of technology in addressing loneliness were also associated with the interrelationship between loneliness and other social problems and campaigns and the concept of loneliness as a symptom or indication of deeper social problems.

Increased online communication was widely represented in the media as potentially harmful in terms of loneliness, through inauthentic connections, addictive use of the internet or even fostering hatred and violence through online communities cut-off from “real” relationships (103). Online life was blamed for the loss of everyday interactions in high streets and public places. Telepresence solutions such as KOMP were found by our participants to be very useful for a wide range of people in a range of settings. However, they also cautioned about relying on digital technologies at the expense of other interventions, such as community navigators and social prescribers, and the need for people to be supported to use technologies (*interview with health service provider*). A lack of relationships for older, isolated people could not be addressed by technological connections alone; if there is no-one to connect to, technology will not help (*interview with charity officer*).

MPs debating the introduction of the government strategy on loneliness introduced their own related local concerns, drawing attention to a whole range of problems including the specific needs of rural constituencies, reduced bus routes, the need for stronger families, funding of libraries and delays in assessing applications for immigration status (69). The introduction by MPs of their constituents' priorities might have been politically expedient but also demonstrated the wide range of other problems associated with loneliness. One stakeholder described loneliness as:

“… a keystone issue I think it's very much like it's the issue that it's both cause and consequence of a whole bunch of other issues so actually if you tackle loneliness effectively not just, not just making someone in contact with people but actually effectively tackle loneliness then you will necessarily act on a whole bunch of other social issues…” (*interview with independent charity and policy consultant*).

There was a breadth of issues related to loneliness throughout our data. The cross-party support for the government strategy allowed loneliness to appear beyond party politics and allowed politicians to address their own ideas about what makes a good society in discussing loneliness. These ranged from concerns with stronger families (69) to austerity and a more individualist society requiring fundamental social change, as Beth Winter MP argued in the House of Commons (104). Technology was frequently represented in our data as having connective power, yet the concept of connected communities required to address loneliness was broader than individual communications, rather, as Kim Leadbetter MP stated, it was about:

“…well-connected, compassionate communities where everyone has a sense of belonging and identity. That tackles a huge range of issues, not just loneliness” (104).

Whereas policy solutions of telepresence technologies promised greater individual connectedness, interpretations of the benefits of technologies centred on their potential to increase participation in collective social life. We interpret these findings, and the rise in loneliness as a campaigning, policy and political issue, as indicating multiple mental models of what makes for good ways to live together in more, or less, successful societies.

**Figure 2:**
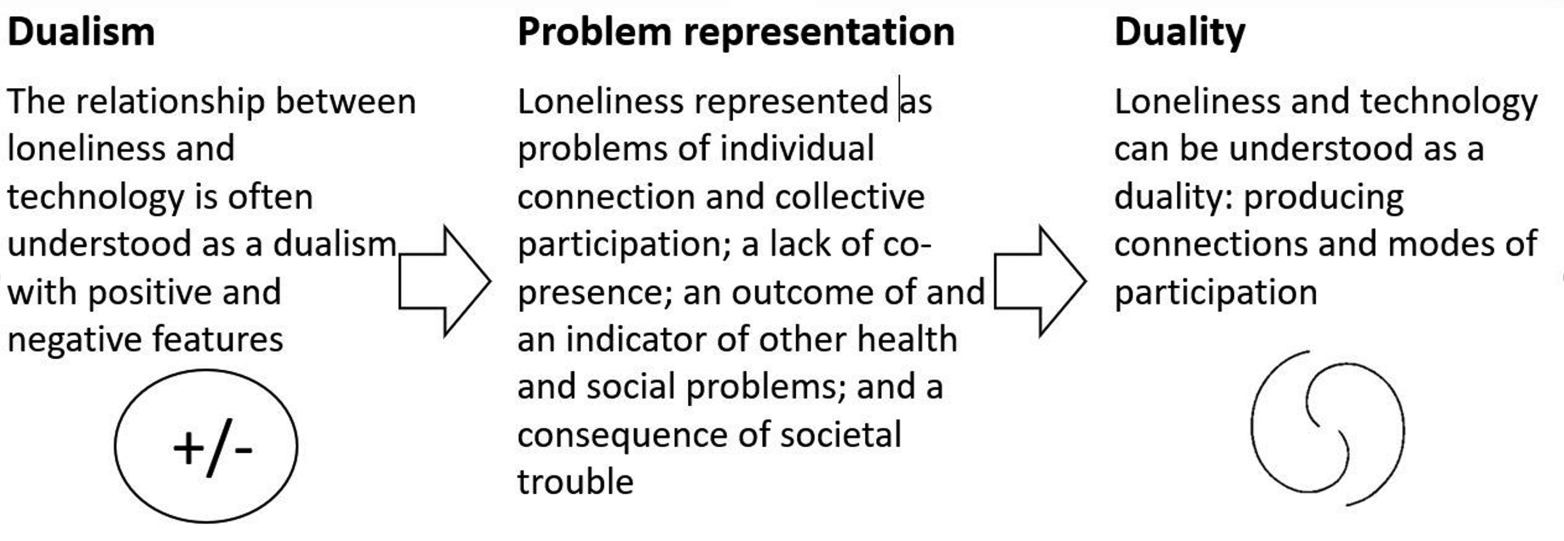
Summary of findings and conclusion.

To sum up our findings, we have investigated the dialectic between loneliness and technology through the lens of practices of adoption of telepresence technologies in the UK. We have found that the relationship between technology and loneliness, frequently represented as a dualism, can also be interpreted as a duality: recursively producing connection and modes of participation that span individual and political perspectives on the causes of loneliness. Our findings and conclusion are summarised in Figure 2.

## Discussion

Our analysis of the policies and practices of adoption of two telepresence technologies in the UK 2020–2022 has provided new insights into the relationship between loneliness and technology and how the problem of loneliness is understood. We have shown, with regards to Bacchi's approach, how the problem of loneliness is represented as: a problem of individual connection and collective participation; a lack of co-presence; an outcome of and an indicator of other health and social problems; and a consequence of societal trouble. We consider here the underpinning assumptions, the silences (what is not represented) and the effects of this representation of the problem, as well as how this representation has been produced, and some divergences and disruptions. Underpinning the representations of loneliness are assumptions about the kinds of connection and participation that are related to youth and old age, the existence of broadly benevolent and available social and technological networks, certain (differing) assumptions about what makes for a good society and the superiority of “real” over virtual presence.

The relationship between technology and loneliness is frequently represented as a dualism; providing both human connection and disconnection. A further dualism emerged from our data of the solution of technology for loneliness in relation to youth and old age. Technology was more frequently represented as troublesome or risky for younger people than older people, in relation to a range of social problems such as bullying in addition to, and interrelated with, loneliness. A moral sense of this dualism was apparent in media coverage of “bad” use of technology exposing younger people to inauthentic or damaging online relationships and “good” use of technology enabling older people to be more connected. Technology, in all shapes and forms including the telephone, was more frequently represented as necessary for older people to address loneliness, leading to support for older people in accessing and using technology. The design and use of the telepresence technologies which were the focus of our study indicated this dualism could be mapped onto the different dimensions of the problem of loneliness: connection and participation. KOMP provided individual connections, indicating that the problem of loneliness for older people interpreted through the design or “script” of KOMP was more akin to “emotional isolation” (59). Assumptions about ageing, as being a more static state and one that includes experiences of loss of emotional relationships (for example through bereavement) are in contrast with assumptions that younger people require participation in social life to undergo the dynamic processes of childhood development. AV1 was designed to enable participation, thus addressing “social isolation” (59). This varied design and use of telepresence technologies indicates the duality of how understandings of loneliness shape technology design and use, and how such technologies shape understandings of loneliness.

Analysis of the relationships between loneliness and technology in our data allowed us to trace representations of certain concerns and aspirations about society. The potential harm of technology as a cause of loneliness, the connections made by policy-makers and campaigners with other social problems and the way in loneliness was understood more widely as a social problem indicated different assumptions about what makes for good, collective social life; how people responded to the problem of loneliness indicated their ideas for what makes for a successful society and indeed their assumptions about the resources and connections that are possible. Providing technology to increase social connections indicates a belief that there are beneficial relationships ready to be made, and assumptions that technology will be readily adopted and connected. The changing context of the pandemic provided new insights through the “national experience of loneliness” (*interview with senior policy maker*) and led to a great increase in use of technologies for social contact, with the adoption of technologies into contexts previously considered unsuitable, but found to be beneficial. Practices of supporting (particularly older) people to use and access technology were necessary when the assumptions about availability of and access to technology were proved to be less well-founded, with the digital divide exposed during the pandemic.

Despite the benefits of technology for connecting people during the conditions of the pandemic, there was a degree of continuity in media representations of technology being at best an inferior form of presence to co-presence and at worst, a source of harm. Pandemic experiences led to greater nuance and appreciation of the importance of maintaining social connections through technology, but perpetuated reservations about the ability of technology-mediated connections to address loneliness and provide satisfactory human connections. An ambivalence was expressed, for example in media articles, about technology as a solution for loneliness relating to the dual nature of the relationship between technology and loneliness. The enduring preference for co-presence creates a limitation for telepresence technologies in addressing loneliness.

Tracing how representations of loneliness have come about in policy and practice shows the legacy of campaigning concerned with increasing community cohesion and strengthening the “bonds of common humanity” (70, p.4). Subsequent campaigns have sought to raise the profile of loneliness as a problem or crisis that must be addressed, mobilising concerns about the costs of loneliness to individuals, society and services which have been associated with a greater focus on loneliness as a public health problem: an epidemic (104).

There is a relative silence in the policy discourse about loneliness as something to be endured; there are few representations of loneliness as an existential component of human existence (9). Instead, it is represented as a social problem to be solved. The consequences of this representation of loneliness include the growth of campaigns and interventions to solve the problem, including technological interventions. Subsequent opportunities arise for growth and profit for organisations and people involved in providing (and indeed researching) solutions. Due to the complexity of adoptions of innovations, which involves an interplay between organisational and policy contexts, adopters and technologies, we have seen work undertaken to influence and change the broader context. Campaigning work to change policy and raise awareness within a range of organisations has emerged from the representations of loneliness as a problem that can be alleviated by technology.

Representations of loneliness as a problem of connection and participation have been reproduced in the development of campaigns and technology aiming to connect people, for example in community activities, volunteering, and online support. Actions aimed at providing additional support to the most deprived communities which are understood as having a weaker “social infrastructure” (*minutes of APPG meeting, 15 June, 2022*) indicate at least a recognition of structural barriers to connection and participation, even if not an alternative representation of the problem itself. However, a greater disruption of this discourse is found in a representation of loneliness as being unevenly distributed in society and having unfair effects, leading to alternative representations and reconceptualisations of the problem of loneliness as being related to poverty and prejudice (98). Telepresence technologies are likely to have limited success in addressing this kind of problem.

Our contribution to the literature on technology and loneliness relates to; the dualism and duality of the relationship between loneliness and technology, the primary of co-presence and the formulation of loneliness as a social rather than an individual problem. There was a degree of interpretive flexibility apparent in relation to both telepresence solutions to loneliness and in understandings of the problem of loneliness. The dynamic nature of the relationship between technology and loneliness analysed by Nowland et al. represents an interpretive flexibility in how technologies are used, the function they serve and what they might mean in addressing (or failing to address) loneliness (12). Internal and external structures (prior knowledge, values and capabilities) shape the use of technologies and their outcomes (105). Similarly, prior knowledge and values shaped how policy-makers and campaigners approached the problem of loneliness. Loneliness was commonly represented as a lack of connection and participation in normal social life, however the reasons why connections and participation were missing (and how they could be found) varied according to diverse ideological and pragmatic positions. In this sense, loneliness as represented in contemporary UK policy and practice is a uniquely social phenomenon; indicative of societal problems. Policy debates are moving from whether to improve connection and participation to considering in what kind of society should we wish people to participate.

### Conclusion

Our study of telepresence technologies has provided new insights into how the problem of loneliness has been represented in UK policy, campaigns, media and practices seeking to address loneliness before and during the COVID-19 pandemic. Loneliness is primarily understood as a painful lack of co-presence which manifests as a problem of human connection and participation in social life. The relationship between loneliness and technology frequently understood as a dualism (offering connection and disconnection) can also be understood as a duality: with use of technology informed by and informing how the problem of loneliness is understood. Our analysis shows how loneliness is not regarded as an existential, or subjective painful experience, integral to human nature, but as a social, societal and policy problem demanding resolution. In this sense, *loneliness* is rewritten as *social isolation* in the social and political handling of the problem.

## Data Availability

Anonymised data supporting this article will be made available by the corresponding author on reasonable request.
